# An Update on the Role of Ubiquitination in Melanoma Development and Therapies

**DOI:** 10.3390/jcm10051133

**Published:** 2021-03-08

**Authors:** Frédéric Soysouvanh, Serena Giuliano, Nadia Habel, Najla El-Hachem, Céline Pisibon, Corine Bertolotto, Robert Ballotti

**Affiliations:** 1 Inserm U1065, C3M, Team 1, Biology, and Pathologies of Melanocytes, University of Nice Côte d’Azur, 06200 Nice, France; frederic.soysouvanh@unice.fr (F.S.); serena.giuliano@univ-cotedazur.fr (S.G.); nadia.habel20@gmail.com (N.H.); celine.pisibon@etu.univ-cotedazur.fr (C.P.); corine.bertolotto@univ-cotedazur.fr (C.B.); 2 Laboratory of Cancer Signaling, University of Liège, 4020 Liège, Belgium; nelhachem@uliege.be; 3 Equipe labellisée Fondation ARC 2019, 06200 Nice, France; 4 Equipe labellisée Ligue Contre le Cancer 2020, 06200 Nice, France

**Keywords:** melanoma, treatment, ubiquitination

## Abstract

The ubiquitination system plays a critical role in regulation of large array of biological processes and its alteration has been involved in the pathogenesis of cancers, among them cutaneous melanoma, which is responsible for the most deaths from skin cancers. Over the last decades, targeted therapies and immunotherapies became the standard therapeutic strategies for advanced melanomas. However, despite these breakthroughs, the prognosis of metastatic melanoma patients remains unoptimistic, mainly due to intrinsic or acquired resistances. Many avenues of research have been investigated to find new therapeutic targets for improving patient outcomes. Because of the pleiotropic functions of ubiquitination, and because each step of ubiquitination is amenable to pharmacological targeting, much attention has been paid to the role of this process in melanoma development and resistance to therapies. In this review, we summarize the latest data on ubiquitination and discuss the possible impacts on melanoma treatments.

## 1. Introduction

Ubiquitination, one of the most conserved protein post-translational modifications, is controlled by the ubiquitin system, a dynamic multifaceted network involved in nearly all aspects of eukaryotic biology. Ubiquitination refers to the covalent attachment of a highly conserved 76-amino acid protein, the ubiquitin, to lysine residues on target proteins. The addition of a single ubiquitin protein or ubiquitin chain is mediated by a cascade of enzymatic reactions carried out by activating, conjugating, and ligating enzymes. The ubiquitination process is well-known to play a key role in protein homeostasis through the control of 26S-mediated proteasome degradation, but also includes nonproteolytic roles, such as receptor internalization, assembly of multiprotein complexes, inflammatory signaling, DNA damage repair, cell death, autophagy, or metabolism [1].

Many proteins, regulated by ubiquitination, control cellular processes relevant to tumorigenesis, such as the modulation of the activity of tumor promoters and suppressors. One of the best-known examples is the RING-type E3 ubiquitin ligase MDM2, a negative regulator of the p53 tumor suppressor [2]. Therefore, ubiquitination enzymes are considered potential therapeutic targets for cancers [3,4]. After the successful clinical application and the approval of proteasomal inhibitors for the treatment of multiple myeloma, substantial progress has been made in understanding the molecular mechanisms of ubiquitin in cancer-relevant processes, and shed light on the therapeutic potential of the ubiquitin system [5].

Even though defects in ubiquitination are not among the most frequent alterations in melanoma, this process has gained more and more attention in the field, as demonstrated by the nearly 300 publications on melanoma and ubiquitination since the last review of Ma et al. in 2017.

## 2. Brief Overview of Melanoma and Its Treatments

Historically, melanoma was a rare cancer, but its incidence has risen rapidly in the last decades. Melanomas represent about 5% of skin cancers, but they are responsible for 90% of deaths from skin cancers. Significant advances in the understanding of melanoma physiopathology have led to the development of new effective treatments for advanced melanomas. Indeed, the identification of BRAF^V600^ mutations in about 50% of melanomas has led to the development of the first targeted therapies (TTs) for patients harboring these mutations. Now, a combination of the BRAF inhibitor (BRAFi) and the MEK inhibitor (MEKi) has shown an overall response rate of up to 70% and has become the standard targeted treatment for BRAF^V600^-mutated melanomas [6]. However, even though long-term responses have been reported, most patients develop resistance and relapse [7,8].

The other breakthrough in melanoma treatment came from immune checkpoint therapies (ICTs), using anti-CTLA4 (Cytotoxic T-Lymphocyte-associated antigen 4) or anti-PD-1 (Programmed cell death protein 1) antibodies. ICTs also showed a dramatic response rate of 44% for anti-PD-1, 20% for anti-CTLA4, and up to 58% when combining both treatments. More interestingly, up to 30% of patients showed a complete and durable response [6,7,9,10,11,12]. Now, the combination of CTLA-4, and PD-1 checkpoint blockades has been proven as a highly efficient treatment for patients with advanced melanomas [13,14]. However, more than half of the patients do not respond to ICT [8,15].

Despite treatment breakthroughs that for the first time improved the survival of patients, the prognosis of metastatic melanoma patients remains unoptimistic. Because of intrinsic or acquired resistance, approximately 50% of patients find themselves in a therapeutic dead-end, which has prompted further research of adjuvant therapies that can improve the efficiency of current standard treatments and patients’ outcomes.

From this perspective, attention has been paid to the regulation of ubiquitination and its consequences on melanoma development and treatments. In this review, we focus on the latest data on ubiquitination in melanoma and discuss the possible impact on melanoma treatments.

## 3. A Glimpse of Ubiquitination Processes

**Figure 1:**
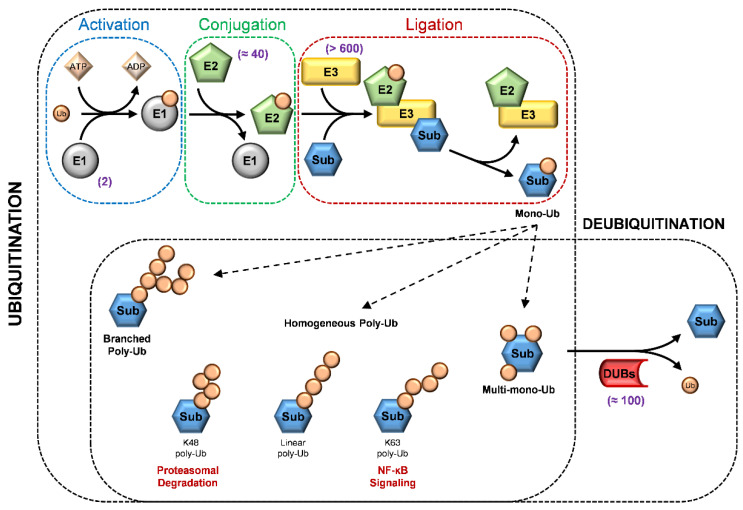
The ubiquitination and deubiquitination process. In the upper panel, three distinct biochemical steps are required for substrate (Sub) ubiquitination: activation performed by activating enzymes (E1s), conjugation by conjugating enzymes (E2s), and ligation by ubiquitin ligases (E3s). In the lower panel, there are different ubiquitin (Ub) modifications and a schematic representation of deubiquitination performed by deubiquitinating enzymes (DUBs). The number of members of each type of enzymes in mammals are highlighted in purple.

Ubiquitination is catalyzed by three distinct biochemical steps: activation, conjugation, and ligation, performed by ubiquitin-activating enzymes (E1s), ubiquitin-conjugating enzymes (E2s), and ubiquitin ligases (E3s), respectively [16]. The first enzyme, E1, catalyzes ubiquitin activation by adenylation of the ubiquitin C-terminus. Subsequently, the mature ubiquitin (Ub) is transferred to the E2-conjugating enzymes. In the final step, the covalent linking of the ubiquitin on the target protein is catalyzed by the E3 ligase, acting as an adapter that recognizes the substrate and mediates the interaction with the Ub-E2. Different combinations of E2 and E3 are possible, providing a wide variability in signal integration and conveying [16] (Figure 1).

E3s are classified into two general classes on the basis of specific protein motifs, the HECT and the RING domain. The HECT domain family is endowed with an intrinsic E3 ligase activity, while the RING-E3 ligases are devoid of enzymatic activity and use the RING domain to bring the E2 ligase to the substrate. The RING domain E3 ligases are divided in single-subunit or multi-subunit proteins, in which the substrate-binding site and RING domain are in the same protein or in a different subunit of the complex [16,17].

The regulation of the various cellular processes is also mediated by different types of ubiquitination. The ubiquitin forms a peptide bond between the ε-NH2 group on the substrate lysyl residues and the C-terminus carboxyl group of Ub (monoubiquitination). It is also possible that multiple lysines in the same substrate are ubiquitinated (multi-monoubiquitination). Ubiquitin, once linked to the target protein, can itself be ubiquitinated on any of its lysine residues (K6, K11, K27, K29, K33, K48, and K63) or its N-terminus methionine (M1), generating poly-Ub chains (polyubiquitination) by the sequential addition of Ub. Poly-Ub chains are homogenous when, during elongation, each ubiquitin is attached to the previous one by the same lysine or methionine or is branched when ubiquitin is linked to a different residue than the previous one. Mixes of homogenous and branched chains also exist [16,18]. These different chain topologies trigger distinct outcomes indicating that the different types of ubiquitin chains can operate as a code, transferring various information to the target proteins [18].

For instance, canonical K48-linked poly-Ub chains are usually the principal signal to target substrates for 26S proteasome proteolysis [19,20]. In contrast, K63-linked chains can act as non-proteolytic signals in several intracellular pathways [21,22]. Ubiquitination can affect protein activity and/or degradation, influencing the regulation of numerous signal pathways. For instance, the NF-κB pathway activation occurs with the degradation of the inhibitor of the NF-κB transcription factor (IκBα). The proteolytic process is initiated by SCFβ-TrCP, a multi-subunit E3 ubiquitin ligase complex [23,24]. In addition, activation of the NF-κB pathway involves the E3 ligase tumor necrosis factor receptor-associated factor 6, TRAF6, which governs the K63-linked polyubiquitination on IKKg (NEMO). This process leads to the release of NF-κB from its inhibitor, and ultimately induces the expression of specific genes [21,22,25].

As ubiquitination is a reversible process, the removal of ubiquitin adducts also plays a key role in cellular biological functions. The deconjugation reactions are catalyzed by deubiquitinating enzymes (DUBs) that precisely cleave the peptide bond between ubiquitin and the target protein after the Gly76 C-terminus of ubiquitin, or between ubiquitins (Figure 1). On the basis of the sequence and domain conservation, DUBs can also be divided into distinct subfamilies, among which ubiquitin-specific proteases (USPs) represent the largest class [26].

Therefore, DUBs, as well as E1, E2, and E3 enzymes, contribute to modulating activation/deactivation, recycling and localizing regulatory proteins, and play important roles in diverse cellular processes, such as DNA repair, apoptosis, cell proliferation, and kinase activation [27].

Compelling evidence established the critical role of ubiquitination in melanoma progression. Indeed, mutations in the deubiquitinating enzyme BRCA1-associated protein 1 (BAP1), in the E3 ligase (or E3 ligase complex) Parkinson protein 2 (PARK2), and in the F-box and WD repeat-containing 7 protein (FBXW7), were shown to favor melanoma development [3]. Moreover, ubiquitination plays an instrumental role in key signaling pathways for melanoma pathogenesis, such as the NF-κB and Wnt/β-catenin pathways [3,28,29,30,31]. In the last three years, numerous studies have increased our knowledge on the involvement of ubiquitination processes in melanoma progression. In the following sections, we focus on the most recent reports dealing with the role of E2, E3, and DUB proteins in melanoma biology.

## 4. E2 Enzyme Involvement in Melanoma Progression

The E2 enzyme family is comprised of nearly 40 members that are involved in the conjugation of Ub or Ub-like molecules to target proteins. The E2 enzymes have been classified into 17 families according to a comprehensive phylogenetic analysis, but broadly fall into 4 different classes: class I contains only the Ub-conjugating (UBC) domain, classes II and III have N- or C-terminus extensions, respectively, and class IV has both N- and C-terminus extensions [32,33]. E2 enzymes are involved in cell cycle progression, DNA repair, apoptosis, and the stimulation of oncogenic signaling pathways (Table 1). In the following section, we describe the roles of E2 enzymes in melanoma progression.

Members of the E2 family were reported to be dysregulated in many cancer types, including melanoma [34]. Indeed, gene expression studies in primary cutaneous melanomas have shown that Ub-conjugating enzyme E2-T (UBE2T) gene expression positively correlates with cell proliferation, tumor progression, and poor prognosis outcome [35]. UBE2T has been involved in the development of various cancers, such as breast cancer through the inhibition of the expression of BRCA1, nasopharyngeal carcinoma by triggering the AKT/Glycogen Synthase Kinase (GSK)-3β/β-catenin pathway, or multiple myeloma as a poor prognosis marker [36,37,38]. A recent study from Dikshit et al. showed that Ub-conjugating enzyme E2-N (UBE2N/Ubc13), a class I E2 enzyme involved in DNA repair, was overexpressed in melanoma cells and played a critical role in melanoma growth and progression both in vitro and in vivo [39]. The systemic inhibition of UBE2N by a selective small molecule, NSC697923, impaired melanoma xenograft growth. In addition to its role in melanoma progression, the authors showed that UBE2N positively regulated and maintained the MEK/FRA1 (Fos-Related Antigen 1)/SOX10 (SRY-related HMG box-containing factor 10) signaling cascade that plays a key role in melanomas [39]. Another E2 enzyme was recently demonstrated to participate in melanoma progression. The Ub-conjugating enzyme E2-C (UBE2C), a key regulator of cell progression, is upregulated in melanomas compared to Spitz nevus [40]. Furthermore, the high mRNA expression level of UBE2C is associated with poor overall survival of patients with melanoma, according to the cancer genome atlas (TCGA) database [41]. The downregulation of UBE2C suppressed melanoma cell growth via the inactivation of the ERK and AKT signaling pathways and the induction of apoptosis. Finally, it was also shown that the knockdown of UBE2C inhibited the growth of xenografted melanoma [41].

Among the E2 family, some members can conjugate small molecules, called Ub-like proteins (UBL), such as small Ub-related modifier (SUMO), neural precursor cell expressed developmentally down-regulated protein 8 (NEDD8), autophagy-related protein 8 (ATG8), autophagy-related protein 12 (ATG12), Ub-related modifier 1 (URM1), Ub-fold modifier 1 (UFM1), human leukocyte antigen F locus adjacent transcript 10 (FAT10), and interferon-stimulated gene 15 (ISG15) [42]. Like ubiquitin, when conjugated to target proteins, UBLs can regulate their activity, stability, subcellular localization, or macromolecular interactions. Furthermore, a crosstalk between ubiquitylation and another post-translational modification, called SUMOylation, has been demonstrated. SUMOylation targets proteins involved in cell cycle regulation, proliferation, apoptosis, and DNA repair. Thus, SUMOylation could impact cancer progression and/or drug responsiveness. As Ub-conjugating enzyme E2-I (UBE2I), also known as Ubc9, is absolutely required for SUMOylation, it has been the subject of numerous studies as a potential target for cancer therapy [43,44]. In melanoma, Ubc9 seems to be upregulated, involved in proliferation, and could play a role in apoptosis evasion in response to chemotherapy treatments [43,45]. Interestingly, it was also demonstrated that Ubc9 interacted with the microphthalmia-associated transcription factor (MITF) [46]. The MITF plays a critical role in melanocyte differentiation, but also melanomagenesis, allowing the transition of melanoma cells between a differentiated-proliferative phenotype and a stem cell-like phenotype [47]. It was described that Ubc9 targets the MITF to the proteasome for degradation, and may favor the transition toward a dedifferentiated and metastatic melanoma phenotype [46]. Whether this regulation involved SUMOylation of ubiquitination remains to be clarified. Nevertheless, SUMOylation of the MITF is paramount for melanoma pathogenesis, as patients with a germline mutation that prevents K316 SUMOylation have an increased risk of developing melanoma [48]. Thus, Ubc9 appears to be a potential target to limit melanoma development.

The ubiquitin-conjugating enzyme E2S (UBE2S) could also play a role in melanoma and be an appealing target. Depletion of UBE2S using short hairpin RNA in melanoma cells resulted in an inhibition of proliferation, a cell cycle arrest, and an increase in apoptosis [49]. In vivo, UBE2S depletion resulted in tumor growth inhibition and the suppression of epithelial-to-mesenchymal transition (EMT)-related markers [49].

RAD6, a ubiquitin-conjugating E2 enzyme, was found to be overexpressed in primary and metastatic melanomas. RAD6 is encoded by two genes, UBE2A (RAD6A) and UBE2B (RAD6B), so Gajan et al. investigated their expression levels in normal melanocytes and melanomas. They demonstrated a selective upregulation of RAD6B in melanoma cells [50]. RAD6B was also linked to Wnt/β-catenin signaling during melanoma progression [51]. RAD6B depletion in metastatic melanoma decreased cell migration, tumor growth, and lung metastasis. The loss of RAD6B also inhibited protein steady-state levels of β-catenin and its transcriptional targets, such as MITF, SOX10, and vimentin. A pathway analysis of transcriptomic data highlighted the implication of various networks, amongst them protein ubiquitination and Wnt signaling [51]. Overall, these findings demonstrate a clear connection between RAD6B and Wnt/β-catenin signaling in melanoma cells and suggest the possibility of targeting RAD6B as a new strategy to treat melanoma.

The involvement of E2 enzymes in many cancer types suggests that specific small chemical inhibitors of E2 enzymes might be valuable in the treatment of cancers, including melanoma. Until now, few E2 enzyme inhibitors have been described [4]. For instance, Leucettamol A and Manadosterols A and B can inhibit Ubc13–UEV1A interaction and block the formation of their complex [4]. CC0651, a small-molecule selective allosteric site inhibitor of the E2 enzyme hCdc34, can block the ubiquitination and degradation of p27 and then inhibit tumor cell proliferation [4]. To date, none of these small molecules have been assessed in the context of melanoma.

**Table 1:** Summary table of identified E2 enzymes and their functional roles in melanoma.

E2 Class		E2s	Roles in Melanoma	Refs
**I**	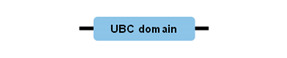	UBE2N UBE2I UBE2B	Overexpressed, proliferation and malignancy Proliferation, apoptosis evasion, sumoylation of MITF Overexpressed, progression and pathogenesis	[39] [44,45,46,47] [50,51]
**II**	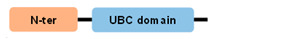	UBE2C	Overexpressed, associated with poor prognosis	[40,41]
**III**	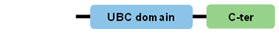	UBE2T UBE2S	Overexpressed, biological role to be determined Proliferation, cell survival, tumor growth, EMT	[35] [49]
**IV**	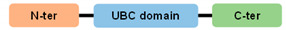			

## 5. E3 Enzyme Involvement in Melanoma

The E3 ubiquitin ligases are considered the most important and specific enzymes in the ubiquitin conjugation machinery. In recent years, E3 ligases received interest as drug targets for their ability to regulate protein stability. Indeed, compared to inhibitors that block the protein degradation through the proteasome, drugs that target E3 ligase are expected to have better selectivity and less toxicity.

**Figure 2:**
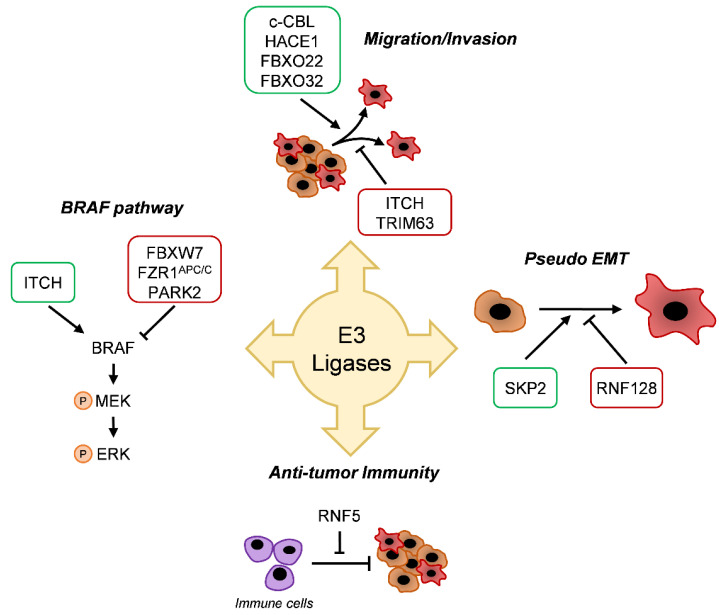
E3-conjugating enzymes implications in melanoma. E3-conjugating enzymes families have been described as players in melanoma progression; for instance, in the BRAF pathway, in the migration and invasion in epithelial-to-mesenchymal transition (EMT) or antitumor immunity. Green boxes are overexpressed in melanoma. Red boxes are underexpressed in melanoma.

Generally, the E3 ligases are classified into four families based on the substrate binding domain: HECT-type, RING-finger-type, U-box-type, and PHD (plant homeodomain)-finger-type ligases. The largest family of E3 ligases is the RING-type SCF (Skp1, Cullins, F-box proteins) E3 family of ligases. SCF complexes consist of four proteins: RING box protein 1 (RBX1) Cullin Protein (CUL), S-phase Kinase-associated Protein 1 (SKP1), which are invariant among SCF complexes, and an F-box protein that varies [52]. Several recent studies have highlighted the critical role of E3 ligase on cancer progression. In line with this, E3 ligase alterations were shown to affect the BRAF and mitogen-activated protein kinase (MAPK) pathways, melanoma migration, and differentiation (Figure 2).

### 5.1. BRAF Pathway

Among the E3 ligase proteins, the F-box and WD repeat-containing 7 protein (FBXW7) was particularly well-studied in cancer progression [53,54,55]. In melanoma, an inactivating mutation of FBXW7 was reported to occur in 8% of melanoma patients [56]. FBXW7 was described as a negative regulator of the mitogen-activated protein kinase (MAPK) pathway by targeting BRAF for degradation. It was demonstrated that FBXW7 loss of function enhanced MAPK activity and promoted resistance to BRAF inhibitors in vitro and in vivo [57]. In parallel, another E3 ligase-targeting BRAF was identified. Wan et al. demonstrated that the APC/C E3 ligase complex and its activator fizzy-related protein 1 (FZR1) both negatively regulated BRAF activity through two distinct mechanisms involving proteolysis and the disruption of BRAF dimerization [58]. Importantly, the authors showed that a loss of FZR1 contributed to Vemurafenib resistance in melanoma cells [58]. These studies showed that E3 ligases can act as tumor suppressors, but some of them are considered as oncogenic players in melanoma progression. That is the case, for instance, of the itchy E3 ubiquitin-protein ligase, or ITCH, which was initially identified as a key enzyme in maintaining a balanced immune response and is strongly associated with autoimmune disease. The role of ITCH in malignancies was unveiled by its ability to tag different substrates for ubiquitination [59]. In melanoma, it was described that ITCH can directly interact with BRAF and promotes its lysine 27-linked polyubiquitination [60]. This atypical non-degradative polyubiquitination of BRAF allows for the recruitment of PP2A that dephosphorylates S365 and disrupts the interaction with the inhibitory scaffold protein 14.3.3, resulting in the sustained activation of BRAF and of its downstream signaling cascade [60].

The E3 ubiquitin ligase PARK2 was defined by Montagnani et al. as a tumor suppressor in malignant melanomas, by uncovering a new mechanism of PARK2 regulation. ELK1 (Ets-like protein 1), a known transcriptional effector of MAPK signaling, represses PARK2, leading to increased melanoma cell proliferation and tumor growth. Moreover, the inhibition of the BRAF-ERK1/2 axis increases PARK2 expression. The authors showed that overexpression of PARK2 in melanoma cells was associated with an antiproliferative effect and cell death in vitro and in vivo [61]. Thus, the reactivation of PARK2 may be an effective approach to counteract melanoma progression.

### 5.2. Migration/Invasion

As previously mentioned, ITCH can tag more than 50 substrates. Thus, it is not surprising that this E3 ligase is involved in several cellular processes like migration and invasion [59,60]. In a recent study, Wang et al. demonstrated that miR-10b, a microRNA targeting ITCH, promoted melanoma progression [62]. The downregulation of miR-10b significantly inhibited cell proliferation, migration, and invasion of melanoma cells in vitro. This work suggests the tumor-suppressing role of ITCH. Of note, FBXW7 was also reported to play a critical role in melanoma cell migration and metastasis [63].

By studying the molecular signature of melanoma, Rambow et al. identified the tripartite motif-containing protein 63 (TRIM63) E3 ligase as a new MITF target gene, and a core gene implicated in cell migration and invasion [64]. In addition, the casitas B-lineage lymphoma (c-CBL), an E3 ubiquitin ligase that was previously associated with acute myeloid leukemia, was found to be strongly expressed in human melanoma compared to benign melanocytic nevi [65]. The knockdown of c-CBL in melanoma cells resulted in decreased proliferation, migration, and spheroid formation. The authors also showed that the knockdown of c-CBL downregulated the FAK-GRB2-SRC signaling pathway, a system known to promote cell growth, proliferation, and motility of normal and neoplastic cells [65].

Recently, our group identified a new player in melanoma biology, the HECT domain and the ankyrin repeat-containing E3 ubiquitin-protein ligase 1 (HACE1). HACE1 is described as a tumor suppressor that catalyzes the degradative ubiquitination of active Rac1 (Ras-related C3 botulinum toxin substrate 1) GTPase [66]. The loss of HACE1 has promoted the progression of numerous cancers, such as breast cancer, hepatocellular carcinoma, or colorectal cancer [67,68,69]. In melanoma, even though expression levels of HACE1 are unchanged between nevi, primary, and metastatic melanoma, the loss of this E3 ligase impairs the migration of melanoma cells [70]. We demonstrated that HACE1 promoted the K27 ubiquitination of fibronectin and favored its secretion. Secreted fibronectin regulates ITGAV (Integrin subunit alpha V) and ITGB1 (Integrin beta-1) expression, globally promoting melanoma cell adhesion and migration. Thus, HACE1 plays a role in melanoma biology [70].

As a part of the SCF complexes, F-box proteins can critically affect cellular processes. A recent study on F-box only protein 22 (FBXO22) by Zheng et al. showed its role as an oncogene and as a potential target in malignant melanoma [71]. The F-box protein FBXO22 is known to specifically interact and induce degradative polyubiquitination of intracellular CD147, also known as basigin [72]. The authors showed a higher expression of FBXO22 in metastatic melanomas compared to normal skin tissue. Moreover, the downregulation of FBXO22 did not impair melanoma cell proliferation in vitro, but affected their migration in vitro and in vivo [71].

Very recently, our group identified FBXO32, a key component of the SCF ubiquitin- protein ligase complexes, as a MITF target, regulating melanoma cell migration and proliferation. Using loss of function approaches, we demonstrated that FBXO32 silencing in melanoma cell lines induced a downregulation of CDK6, a cell cycle protein promoting proliferation, and an upregulation of SMAD7, an inhibitor of the TGF-β pathway linked to cell migration. At the molecular level, FBOX32 seemed to interact with BRG1 (Brahma-related gene-1), a chromatin-remodeling protein [73]. This interaction could modulate the gene expression responsible for melanoma progression.

### 5.3. Differentiation

Epithelial-to-mesenchymal transition is a complex biological process by which immotile epithelial cells switch to motile mesenchymal cells. Mesenchymal cells are more invasive, more aggressive, and frequently display resistance to therapies. Melanocytes derive from neural crest epithelium through a first EMT. However, because they reside in the epidermis, melanocytes retain some epithelial cell attributes such as the expression of E-cadherin. Melanoma can also retain a certain degree of the epithelial phenotype, but can further switch to a more mesenchymal phenotype with exacerbated invasive properties and a strong resistance to TTs [74].

Over the last decade, numerous studies have involved ubiquitination in the regulation of EMT. The role of the F-box family was particularly well-studied in various types of cancer [75]. For instance, FBXW7 can suppress cell migration and invasion by negatively regulating the transcription factor SNAIL in human non-small cell lung cancer (NSCLC) [54]. As FBXW7 is frequently downregulated in cancer cell lines, functional studies revealed that a higher level of FBXW7 dramatically inhibited migration and invasiveness of renal cell carcinoma [76]. SKP2, a member of the F-box/LRR-repeat protein (FBXL) subfamily, is known to participate in degradative ubiquitination of the cyclin-dependent kinase inhibitor p27^kip1^, a negative cell cycle regulator. Therefore, the inhibition of SKP2 in diverse cancers, including cutaneous [77,78] and uveal melanomas [79,80], was reported to inhibit cell proliferation in vitro and suppress tumor development in vivo. In 2014, a study revealed that the levels of SKP2 were elevated by TGF-β1 treatment in human melanoma cells [81]. It is known that TGF-β1 induces EMT [82]. Increased levels of SKP2 were accompanied with AKT and c-Myc activation during EMT [81]. Recently, the RING finger protein 128 (RNF128), an E3 ligase from the RING family, was described to favor melanoma development by inducing EMT [83]. The authors showed that RNF128 was downregulated in melanoma compared to peripheral normal tissue. The downregulation of RNF128 promoted melanoma cell proliferation in vitro and in vivo, through the degradative ubiquitination of CD44 and cortactin (CTTN). These two factors can activate the Wnt pathway, previously described as a critical axis involved in EMT and stemness in melanoma [75,83].

### 5.4. Antitumor Immunity

An increasing number of studies reveal a closer connection between the microbiota and cancer immunity, particularly the capability of the microbiota to regulate the expression of the cancer immune checkpoints [84]. Recently, Li et al. demonstrated that an alteration of intestinal microbiota in Rnf5^−/−^ mice may have a role in antitumor immunity [85]. RING finger protein 5 (RNF5) is a E3 ubiquitin ligase, localized in the endoplasmic reticulum (ER) membrane and implicated in numerous cell processes, including the ER quality control system, through ubiquitination of misfolded proteins. Li et al. showed that RNF5 regulated the antitumor immunity and controlled melanoma tumor growth. A significant reduction of the unfolded protein response (UPR) components was seen in the response to RNF5 deletion, related with inflammasome increase, recruitment and activation of dendritic cells and T-cells, and the reduced expression of antimicrobial peptides (AMPs) in intestinal epithelial cells. Decreased AMPs might cause imbalance in the gut microbiota composition and promote a pro-inflammatory tumor micro-environment. The importance of the gut microbiota in the control of tumor growth in Rnf5^−/−^ mice was confirmed by an antibiotic cocktail treatment that prevented tumor growth inhibition. Their data demonstrated that RNF5 loss, linked with an altered UPR signaling, coincided with variations in gut microbiota composition, activation of antitumor immunity, and consequently, efficient melanoma growth inhibition [85].

## 6. Deubiquitination and Melanoma

Protein ubiquitination processes can be reversed by deubiquitinating enzymes, which cleave the isopeptide bond between ubiquitin and its substrate [86]. These enzymes fall into two main categories: the cysteine proteases comprising the ubiquitin C-terminus hydrolases (UCHs), ubiquitin-specific proteases (USPs), ovarian tumor proteases (OTUs), Machado–Josephin domain proteases (MJDs), and the metalloprotease Jab1/MPN/Mov34 (JAMM) domain containing metalloisopeptidase [87]. Dysregulation of the DUB, and the consequent alteration of the ubiquitin system, are involved in the increase of the oncogene effects and/or decrease in the tumor suppressor activity in cancers in general, and in melanoma specifically.

### 6.1. Tumor Suppressors

BAP1 (BRCA-associated protein 1) is a nuclear deubiquitinase belonging to UCH family. BAP1 acts as tumor suppressor and is involved in many crucial cellular processes [88]. Germline mutations of BAP1 have been associated with hereditary predisposition to multiple cancers, including uveal melanoma (UM) and cutaneous melanoma (CM). Individuals who carry the mutated BAP1 gene develop melanocytic lesions later in life, and some of those benign lesions can transform into cutaneous melanomas [89]. BAP1 has been demonstrated to be involved in DNA damage and deubiquitination of the histone H2A, a histone family related to cell differentiation and organism development, and associated with cancer [90,91]. More recently, Webster et al. showed that BAP1 deletion in melanocytes cooperated with the oncogenic form of BRAF to promote melanoma growth in mice [92]. Interestingly, the loss of BAP1 was associated with apoptosis in a large set of cell types, but not in melanocytes and mesothelial cells, where its inactivation favored tumorigenesis, demonstrating a cell-specific tumor suppressor function of BAP1 [93]. BAP1 was also reported to exert its tumor suppressor function through the Hippo pathway that plays a key role in uveal melanoma [94].

Despite this, a recent meta-analysis based on TCGA dataset described opposite roles of BAP1 in survival of uveal and cutaneous melanoma. This analysis showed that low BAP1 mRNA was associated with a better overall survival (OS) in CM patients, particularly in older patients, in contrast with a poor OS in UM patients [95]. This analysis was in contrast with preceding studies, where the depletion of BAP1 expression indicated a worse outcome in CM patients [92,96].

Another deubiquitinase that is well-known as tumor suppressor is the cylindromatosis (CYLD) tumor suppressor protein, a UCH deubiquitinase that predominantly removes K63- and M1-linked chains from target proteins [97]. CYLD was shown to deubiquitinate different substrates, such as the proto-oncogene BCL-3 (B-cell chronic lymphatic leukemia protein 3), preventing its nuclear translocation and accumulation, which is associated with activation of NF-κB-dependent gene transcription and cell proliferation [98].

CYLD is suppressed in human melanoma cells, by the transcription factor SNAIL1. Loss of CYLD stimulates cellular proliferation, migration, and invasion by triggering BCL-3 nucleus translocation and activation of cyclin D1 and N-cadherin [99].

Recently, the role of CYLD was investigated in a murine model (Grm1) for spontaneous melanoma development [100]. The authors demonstrated that CYLD-knockout mice displayed increased tumor growth compared to wild-type mice. CYLD-deficiency appeared to favor lymphatic angiogenesis [100].

### 6.2. Tumor Promoters

Another deubiquitinase family critical for cancer progression is the ubiquitin-specific peptidases (USPs) family. USP deubiquitinases are involved in various aspects of the tumorigenesis process, including the regulation of transcription factors, apoptosis-related factors, DNA repair activity, histone modifications, and cell cycle progression [87,101].

The ubiquitin-specific protease USP4 appears as a regulator of different cellular pathways and targets a variety of substrates. It was found that the expression of the USP4 was upregulated in melanoma tissues and cell lines [102]. Impairing the expression of USP4 inhibits the invasive and migratory ability of melanoma cells. This phenotype correlates with a downregulation of N-cadherin and upregulation of E-cadherin, suggesting that EMT could be reversed. These data indicate that USP4 may act as an oncogene [102].

Very recently, Guao et al. showed that Spautin-1, a small-molecule autophagy inhibitor, capable of inhibiting the deubiquitinating activity of USP10 and USP13, induced cell cycle arrest in G2 phase and increased cell apoptosis in melanoma cell lines [102]. These results reveal the potential interest of USP10/USP13 targeting by Spautin-1 as an anti-melanoma strategy [103].

## 7. Ubiquitination and Resistance

Despite the conspicuous clinical response of BRAF-mutated melanoma to BRAF inhibitors and the dramatic response rate of immune checkpoint therapies, the prognosis of melanoma patients remains unfavorable, mainly due to the development of drug resistance. In this last part of the review, we discuss the involvement of the ubiquitination system toward melanoma drugs and immune checkpoint therapy resistance.

### 7.1. Drugs Resistance

In the past few years, some actors have been identified as crucial players in melanoma drug resistance, like NEDD4 [103]. NEDD4 belongs to a subfamily of HECT E3 ligases and mediates substrate ubiquitination and proteasomal degradation, as well as receptor-mediated endocytosis. NEDD4 exhibits an oncogenic function. Indeed, the inhibition of NEDD4 ubiquitination activity promotes the PTEN stabilization, which can induce an antiproliferative response in melanoma [104]. Very recently, Yang et al. demonstrated that the voltage-dependent anion-selective channel (VDAC) 2 and 3, during erastin-induced ferroptosis in melanoma cells, were ubiquitinated by NEDD4 and sent to degradation. The knockdown of NEDD4 increased the VDAC2/3 protein level, with a consequent improvement of erastin sensitivity in melanoma cell lines and in the mice xenograft model [105]. These results uncover the crucial role of NEDD4 in the negative regulation of erastin-induced ferroptosis in melanoma.

In 2015, Kim et al. identified the ubiquitin ligase RNF125 as a crucial component of the innate and adaptive resistance in BRAFi-resistant melanomas [106]. RNF125 is a RING-type E3 ubiquitin ligase that acts as a positive regulator in T-cell activation, but a negative one in the antiviral innate immunity [107,108]. A decreased level of RNF125 transcript in BRAFi-resistant melanoma cells conferred a growth advantage in the presence of BRAFi. The inhibition of Janus kinase 1 (JAK1) activity due to ubiquitination by RNF125 decreased EGFR expression at the transcriptomic and protein levels, overcoming BRAFi resistance in melanoma cells [106]. These data suggest an important role of RNF125 in reducing the growth of BRAFi-resistant melanoma by the dysregulation of JAK and EGFR.

The same team recently reported that RNF4 promotes tumorigenesis and confers resistance to targeted therapies in melanoma [109]. RNF4 is a SUMO-dependent E3 ubiquitin ligase implicated in cancer that regulates the tumorigenesis of melanoma. Mechanistically, RNF4 seems to bind, ubiquitinate, and increase the expression of eIF2α. Moreover, the author showed that the RNF4–eIF2α axis plays an important role in the resistance of melanoma cells toward BRAFi [109].

As part of the RING-type family and HECT family, the F-box E3 ligase family member FBXO42 was also recently described as involved in the resistance of melanoma to targeted therapies [110]. Using the CRISPR–Cas9-mediated genome-wide screen, the authors identified FBXO42 loss as a driver of trametinib (MEK inhibitor) resistance in NRAS-mutated melanoma.

Data in the literature indicate that ubiquitin-specific peptidases (USPs), the main members of the deubiquitinase family, are involved in DNA damage repair activity, suggesting that USPs may be linked to drug resistance during cancer treatment. USPs are investigated as possible targets to develop inhibitors for cancer prevention [101]. Recently, a study described how the depletion of USP28 favored resistance to BRAF inhibitor therapies. USP28 deubiquitinated and stabilized FBXW7, a component of the SCF ubiquitin ligase complex that controls the degradation of BRAF [111]. Therefore, USP28 depletion increased BRAF protein levels and melanoma cell resistance to BRAF inhibitors [57]. These results show that USP28 is a key factor in ERK pathway activation and in resistance to BRAF inhibitors in vitro and in vivo.

Increased activity of USP14, a proteasome-associated DUB, was observed in melanoma cells and in melanoma patients compared to normal skin and nevi b-AP15, a selective USP14 inhibitor; the USP14 reduced proliferation of melanoma cells independent of the mutational cell status. The selective inhibitor b-AP15 also showed anti-melanoma activity in a mouse model of a BRAFi-resistant tumor, suggesting that USP14 is a possible target in melanoma with acquired resistance to targeted therapies [112].

A genetic screen of the whole-genome shRNA library led to the identification of two negative regulators of resistance to Vemurafenib in BRAF^V600E^-expressing melanoma cells: neurofibromin 1 (NF1) and CUL3 [113]. The authors showed that loss of CUL3, a core component of the SCF E3 ubiquitin ligase complex, activates Rac1 leading to MAPKi resistance. Inhibition of the SRC family could reverse resistance induced by CUL3 depletion via the inactivation of the Rac1 protein [113]. These data highlight the SRC-Rac1 signaling axis as a new mechanism implicated in BRAFi resistance.

### 7.2. Resistance to ICTs

As for targeted therapies, patients with melanoma also develop resistance to immunotherapies. Antibody inhibitors against PD-1 or its ligand (PD-L1) have become commonly used to treat various types of cancer [114]. Recently, Meng et al. described a regulatory mechanism of PD-1 and demonstrated its critical role in antitumor immunity [115]. FBXO38, a component of a SCF E3 ligase complex, was reported to induce K48-linked polyubiquitination of PD-1 and cause its proteasomal degradation. In vivo experiments in mice showed that FBXO38 knockdown led to faster tumor progression along with a higher PD-1 expression level. This study highlights the clinical potential of FBXO38 as it offers an alternative method to block the PD-1 pathway [115].

In the same way, Otubain 1 (OTUB1) is a deubiquitinase member of the ovarian tumor (OTU) domain family that specifically cleaves K48-linked polyubiquitin chains, regulates many cancer associated signaling pathways, and has a critical role in cancer initiation and progression [116]. In 2019, it was shown that OTUB1 is a crucial controller of the activation and function of CD8 T-cells and Natural Killer (NK) cells in immune responses against cancer [117]. Indeed, the deletion of OTUB1 in T-lymphocytes or NK cells increased their anti-melanoma activity, establishing its key role as a regulator of antitumor immunity and as a potential target to improve immunotherapy [117].

Very recently, Scortegagna et al. identified the role of the E3 ubiquitin ligase SIAH2 (Seven in absentia homolog 2) in the regulation of T-regulatory (Treg) cells [118]. In Siah2^−/−^ nude mice inoculated with melanoma cells, tumor-infiltrating Treg cells were dramatically less proliferative, leading to the inhibition of tumor growth, compared to Treg from wild-type mice. Moreover, the tumor growth was drastically reduced when Siah2^−/−^ mice were challenged with anti-PD-1 treatment [118]. The authors concluded that SIAH2 controls Treg-cell recruitment and its loss in the host sensitizes melanoma to anti-PD-1 treatment. Thus, targeting SIAH2 could be beneficial to impair melanoma growth and development. The Ronai lab is currently developing SIAH1/2 inhibitors, able to affect melanoma cell viability, that could further be used in combination with targeted or immune checkpoint therapies [119].

Furthermore, in the immunotherapy context, Mezzadra et al. recently demonstrated that loss of chemokine-like factor (CKLF)-like MARVEL transmembrane domain containing family member 6 (CMTM6) decreased PD-L1 protein levels in melanoma cells [120]. At molecular level, the authors showed that CMTM6 interacted with PD-L1 and protected it from degradative ubiquitination. In agreement with this observation, CMTM6, by increasing PD-L1-expression, enhances the ability of tumor cells to inhibit the function of T-cells [120]. Thus, targeting CMTM6 to increase PD-L1 ubiquitination and degradation has a potential value as a therapeutic strategy to improve the immune response of melanoma cells (Table 2).

## 8. Conclusions and Future Directions

An increasing number of studies have demonstrated the critical role of ubiquitination in cancer development, progression, and resistance to therapies, and this also holds true in melanoma. As a major post-translational modification, ubiquitination controls the expression and function of proteins. This level of regulation should be considered in addition to gene expression levels to clearly understand the processes of melanoma development.

Since ubiquitination is involved in most of the cellular processes that are deregulated in tumor cells, targeting ubiquitination has appeared to be a rational therapeutic strategy in cancers. However, the pleiotropic role of ubiquitination also raises concerns about possible adverse effects, unless an enzyme that is specifically expressed in the considered neoplasm is targeted.

Proteasome inhibitors (Bortezomib, Carfilzomib, Ixazomib) were one of the first drugs, which interfere with ubiquitination processes, that were successfully used in clinical trial for multiple myeloma [121]. With an overall response rate of 23.7% (18.7–29.4), and a median duration of response of 7.8 months (5.6–9.2), Carfilzomib was shown to be a safe and effective treatment option for patients with relapsed multiple myeloma refractory, when compared to Bortezomib, Thalidomide, or Lenalidomide [122]. The first reversible and orally administered proteasome inhibitor, Ixazomib, was approved by the FDA in 2015 [123]. Ixazomib showed an overall response rate of 27% at the maximum tolerated dose. It appears to be less toxic, with an excellent tolerability, when compared to Bortezomib [123]. Of note, no beneficial effects were observed in metastatic malignant melanoma patients treated with Bortezomib [124]. Basically, each step of the ubiquitination process can be pharmacologically targeted. Indeed, several component targeting E1 ubiquitin-activating enzymes have been described. Among them, Pevonedistat is being used in clinical trials for acute myeloid leukemia and melanoma [125]. Because of the absence of a classical druggable site, few inhibitors of E2 ubiquitin-conjugating enzymes have been described so far, and none are in clinical trial [126]. Concerning the E3 ligases, as they are considered as the pivotal enzyme in the ubiquitination process, efforts have allowed the discovery of specific inhibitors. For instance, an inhibitor of MDM2, a key regulator of p53 stability, has been approved in clinic for liver and pancreatic cancer [4]. Among the FDA-approved E3 modulators, Thalidomide and Lenalidomide have shown no significant responses in their respective phase II studies [127,128].

Finally, deubiquitinating enzymes that reverse ubiquitination are also the target of inhibitors. Pimozide, an USP1 inhibitor, is in clinical trial for glioblastoma [129].

However, the successful use of ubiquitination process inhibitors has probably been limited because of the lack of specificity of the drugs used. Recently, approaches hijacking the ubiquitin-proteasome system (UPS) have emerged to overcome specificity and redundancy problems. The first one uses ubiquitin variants (UbVs) that were designed to improve potency and specificity toward UPS enzymes. However, the delivery of these engineered proteins to the cells remains challenging. The second one is an approach gaining increasing interest because it can potentially target 97% of the reputable, undruggable proteins. This approach, called proteolysis-targeting-chimera (PROTAC) uses heterobifunctional compounds that foster the formation of a complex between an E3 ligase and the target protein, promoting ubiquitination and degradation of the latter. Virtually, this approach might be used to target and destroy any protein essential in tumor development.

Today, the few clinical trials using inhibitors of the UPS have shown no clear objective benefits in patients with melanoma. Nevertheless, recent reports indicating that ubiquitination affects responses to targeted and immune therapies might prompt the evaluation of inhibitors of ubiquitination process in combination with current treatments. The PROTAC approach, specifically targeting the epigenetic processes or key oncogenic pathways, also deserves further investigation in the context of melanoma [130,131].

## Figures and Tables

**Table 2:** Deubiquitinating enzymes and their roles in melanoma progression.

Family	Gene	Substrate	Pathway	Function in melanoma		Refs
UCH	**BAP1**	Histone H2A	DNA double-strand break repair	Cell differentiation	Tumor suppressor	[83,84,85,86,87,88,89]
	**CYLD**	BCL-3	N-cadherin expression	Proliferation, migration, invasion and lymph angiogenesis	Tumor suppressor	[93]
		-	Angiogenesis			[95]
USP	**USP4**	-	EMT	Migration and invasion	Tumor promoter	[97]
	**USP10/13**	p53	p53	Proliferation	Tumor promoter	[98,99]
	**USP14**	Proteasome substrates	UPS	Proliferation	Resistance promotion	[109]
	**USP28**	Fbw7	MAPK	BRAF inhibitor resistance	Resistance prevention	[108]
OTU	**OTUB1**	UBE2N	DNA double-strand breaks repair	Initiation and progression	Tumor promoter	[113]
		AKT	CD8 T cells and NK cells activation	Immune cell activation		[114]

UCH: ubiquitin C-terminus hydrolase; BAP1: BRCA-associated Protein 1; CYLD: cylindromatosis tumor suppressor protein; OTU: ovarian tumor protease; USP: ubiquitin-specific protease; EMT: epithelial-to-mesenchymal transition; UPS: ubiquitin-proteasome system.

## Data Availability

No new data were created or analyzed in this study. Data sharing is not applicable to this article.

## Abbreviations

AKT: A Serine/Threonine Kinase; AMPs: Antimicrobial Peptides; APC/C: Anaphase-Promoting Complex/Cyclosome; ATG: Autophagy-related protein; BAP1: BRCA1-Associated Protein 1; BCL3: B-cell chronic lymphatic leukemia protein 3; BRAF: Rapidly Accelerated Fibrosarcoma B-type; BRCA1: Breast Cancer 1; BRG1: Brahma-Related Gene-1; c-CBL: Casitas B-lineage lymphoma; CD147: Cluster of Differentiation 147; CDK6: Cyclin Dependent Kinase 6; CKLF: Chemokine-Like Factor; CM: Cutaneous Melanoma; CMTM6: Chemokine-like factor (CKLF)-like MARVEL Transmembrane domain containing family member 6; c-Myc: Avian Myelocytomatosis virus oncogene cellular homolog; CRISPR: Clustered Regularly Interspaced Short Palindromic Repeats; CTLA4: Cytotoxic T-Lymphocyte-associated antigen 4; CTTN: Cortactin; CUL: Cullin Protein; CYLD: Cylindromatosis tumor suppressor protein; DUBs: Deubiquitinating enzymes; EGFR: Epidermal Growth Factor Receptor; ELK1: Ets-Like protein 1; EMT: Epithelial-to-Mesenchymal Transition; ER: Endoplasmic Reticulum; ERK: Extracellular signal-Regulated Kinase; FAT10: human leukocyte antigen F locus Adjacent Transcript 10; FBXW7: F-box and WD repeat-containing 7 protein; FBXL: F-box/LRR-repeat protein; FBXO: F-box Only protein; FRA1: Fos-Related Antigen 1; FZR1: Fizzy-Related protein 1; GSK: Glycogen Synthase Kinase; HACE1: ankyrin repeat-containing E3 ubiquitin-protein ligase 1; HECT: Homology to E6-AP C-Terminus; ICTs: Immune Checkpoint Therapies; ITCH: Itchy E3 ubiquitin-protein ligase; ITGAV: Integrin subunit Alpha V; ITGB1: Integrin Beta-1; ISG15: interferon-stimulated gene 15; JAK1: Janus Kinase 1; JAMM: Metalloprotease Jab1/MPN/Mov34; MAPK: Mitogen-Activated Protein Kinase; MDM2: Mouse Double Minute 2; MEK: MAPK/Erk1 Kinase; MITF: Microphthalmia-associated Transcription Factor; MJDs: Machado–Josephin domain proteases; NEDD: Neural precursor cell Expressed Developmentally Down-regulated protein; NF1: Neurofibromin 1; NK: Natural Killer; NRAS: Neuroblastoma; RAS: viral oncogene homolog; PARK2: Parkinson Protein 2; PD1: Programmed cell death protein 1; PHD: Plant Homeodomain; PP2A: Protein serine/threonine Phosphatase 2A; PROTAC: proteolysis-targeting-chimera; PTEN: Phosphatase and Tensin homolog; OTUs: Ovarian Tumor proteases; OTUB1: Otubain 1; OS: overall survival; RBX1: RING box protein 1; RING: Really Interesting New Gene; RNF: RING finger protein; TGF: Transforming Growth Factor; TRAF6: Tumor Necrosis Factor Receptor-associated Factor 6; TRIM63: Tripartite Motif-containing protein 63; TTs: Targeted Therapies; Treg: T-regulatory; SCF: Skp1, Cullins, F-box proteins; SIAH2: Seven In Absentia Homolog 2; SKP: S-phase Kinase-associated Protein; SMAD7: Mothers against decapentaplegic homolog 7; SOX10: SRY-related HMG box-containing factor 10; SUMO: Small Ub-related Modifier; Ub: Ubiquitin; UBC: Ub-conjugating; UBE: Ub-conjugating enzyme E; UBL: Ub-like proteins; UbVs: Ubiquitin Variants; UCHs: Ubiquitin C-terminus Hydrolases; UEV1A: Ubiquitin-conjugating Enzyme Variant 1A; UFM1: Ub-Fold Modifier; UM: Uveal Melanoma; UPR: Unfolded Protein Response; UPS: Ubiquitin-Proteasome System; URM1: Ub-Related Modifier 1; USPs: Ubiquitin-Specific Proteases; VDAC: Voltage-Dependent Anion-selective Channel; Wnt: Wingless-related integration site.

## References

[1] Swatek K.N., Komander D. (2016). Ubiquitin modifications. Cell Res..

[2] Senturk E., Manfredi J.J. (2012). Mdm2 and tumorigenesis: Evolving theories and unsolved mysteries. Genes Cancer.

[3] Ma J., Guo W., Li C. (2017). Ubiquitination in melanoma pathogenesis and treatment. Cancer Med..

[4] Deng L., Meng T., Chen L., Wei W., Wang P. (2020). The role of ubiquitination in tumorigenesis and targeted drug discovery. Signal Transduct. Target. Ther..

[5] Popovic D., Vucic D., Dikic I. (2014). Ubiquitination in disease pathogenesis and treatment. Nat. Med..

[6] Long G.V., Stroyakovskiy D., Gogas H., Levchenko E., de Braud F., Larkin J., Garbe C., Jouary T., Hauschild A., Grob J.-J. (2015). Dabrafenib and trametinib versus dabrafenib and placebo for Val600 BRAF-mutant melanoma: A multicentre, double-blind, phase 3 randomised controlled trial. Lancet.

[7] Long G.V., Stroyakovskiy D., Gogas H., Levchenko E., de Braud F., Larkin J., Garbe C., Jouary T., Hauschild A., Grob J.J. (2014). Combined BRAF and MEK inhibition versus BRAF inhibition alone in melanoma. N. Engl. J. Med..

[8] LoRusso P.M., Schalper K., Sosman J. (2019). Targeted therapy and immunotherapy: Emerging biomarkers in metastatic melanoma. Pigment Cell Melanoma Res..

[9] Hauschild A., Grob J.-J., Demidov L.V., Jouary T., Gutzmer R., Millward M., Rutkowski P., Blank C.U., Miller W.H., Kaempgen E. (2012). Dabrafenib in BRAF-mutated metastatic melanoma: A multicentre, open-label, phase 3 randomised controlled trial. Lancet.

[10] McArthur G.A., Chapman P.B., Robert C., Larkin J., Haanen J.B., Dummer R., Ribas A., Hogg D., Hamid O., Ascierto P.A. (2014). Safety and efficacy of vemurafenib in BRAF(V600E) and BRAF(V600K) mutation-positive melanoma (BRIM-3): Extended follow-up of a phase 3, randomised, open-label study. Lancet. Oncol..

[11] Hodi F.S., O’Day S.J., McDermott D.F., Weber R.W., Sosman J.A., Haanen J.B., Gonzalez R., Robert C., Schadendorf D., Hassel J.C. (2010). Improved survival with ipilimumab in patients with metastatic melanoma. N. Engl. J. Med..

[12] Wolchok J.D., Chiarion-Sileni V., Gonzalez R., Rutkowski P., Grob J.-J., Cowey C.L., Lao C.D., Wagstaff J., Schadendorf D., Ferrucci P.F. (2017). Overall Survival with Combined Nivolumab and Ipilimumab in Advanced Melanoma. N. Engl. J. Med..

[13] Sharma P., Hu-Lieskovan S., Wargo J.A., Ribas A. (2017). Primary, Adaptive, and Acquired Resistance to Cancer Immunotherapy. Cell.

[14] Kruger S., Ilmer M., Kobold S., Cadilha B.L., Endres S., Ormanns S., Schuebbe G., Renz B.W., D’Haese J.G., Schloesser H. (2019). Advances in cancer immunotherapy 2019—Latest trends. J. Exp. Clin. Cancer Res..

[15] Schachter J., Ribas A., Long G.V., Arance A., Grob J.J., Mortier L., Daud A., Carlino M.S., McNeil C., Lotem M. (2017). Pembrolizumab versus ipilimumab for advanced melanoma: Final overall survival results of a multicentre, randomised, open-label phase 3 study (KEYNOTE-006). Lancet.

[16] Pickart C.M. (2001). Mechanisms underlying ubiquitination. Annu. Rev. Biochem..

[17] Satija Y.K., Bhardwaj A., Das S. (2013). A portrayal of E3 ubiquitin ligases and deubiquitylases in cancer. Int. J. cancer.

[18] Komander D., Rape M. (2012). The ubiquitin code. Annu. Rev. Biochem..

[19] Chau V., Tobias J.W., Bachmair A., Marriott D., Ecker D.J., Gonda D.K., Varshavsky A. (1989). A multiubiquitin chain is confined to specific lysine in a targeted short-lived protein. Science.

[20] Pickart C.M., Fushman D. (2004). Polyubiquitin chains: Polymeric protein signals. Curr. Opin. Chem. Biol..

[21] Deng L., Wang C., Spencer E., Yang L., Braun A., You J., Slaughter C., Pickart C., Chen Z.J. (2000). Activation of the IkappaB kinase complex by TRAF6 requires a dimeric ubiquitin-conjugating enzyme complex and a unique polyubiquitin chain. Cell.

[22] Wang C., Deng L., Hong M., Akkaraju G.R., Inoue J., Chen Z.J. (2001). TAK1 is a ubiquitin-dependent kinase of MKK and IKK. Nature.

[23] Winston J.T., Strack P., Beer-Romero P., Chu C.Y., Elledge S.J., Harper J.W. (1999). The SCFbeta-TRCP-ubiquitin ligase complex associates specifically with phosphorylated destruction motifs in IkappaBalpha and beta-catenin and stimulates IkappaBalpha ubiquitination in vitro. Genes Dev..

[24] Margottin-Goguet F., Hsu J.Y., Loktev A., Hsieh H.M., Reimann J.D.R., Jackson P.K. (2003). Prophase destruction of Emi1 by the SCF(betaTrCP/Slimb) ubiquitin ligase activates the anaphase promoting complex to allow progression beyond prometaphase. Dev. Cell.

[25] Walsh M.C., Lee J., Choi Y. (2015). Tumor necrosis factor receptor- associated factor 6 (TRAF6) regulation of development, function, and homeostasis of the immune system. Immunol. Rev..

[26] Yuan T., Yan F., Ying M., Cao J., He Q., Zhu H., Yang B. (2018). Inhibition of Ubiquitin-Specific Proteases as a Novel Anticancer Therapeutic Strategy. Front. Pharmacol..

[27] Wilkinson K.D. (2009). DUBs at a glance. J. Cell Sci..

[28] Fuchs S.Y., Spiegelman V.S., Kumar K.G.S. (2004). The many faces of beta-TrCP E3 ubiquitin ligases: Reflections in the magic mirror of cancer. Oncogene.

[29] Liu J., Suresh Kumar K.G., Yu D., Molton S.A., McMahon M., Herlyn M., Thomas-Tikhonenko A., Fuchs S.Y. (2007). Oncogenic BRAF regulates beta-Trcp expression and NF-kappaB activity in human melanoma cells. Oncogene.

[30] Santra M.K., Wajapeyee N., Green M.R. (2009). F-box protein FBXO31 mediates cyclin D1 degradation to induce G1 arrest after DNA damage. Nature.

[31] Lee E.K., Lian Z., D’Andrea K., Letrero R., Sheng W., Liu S., Diehl J.N., Pytel D., Barbash O., Schuchter L. (2013). The FBXO4 tumor suppressor functions as a barrier to BRAFV600E-dependent metastatic melanoma. Mol. Cell. Biol..

[32] Valimberti I., Tiberti M., Lambrughi M., Sarcevic B., Papaleo E. (2015). E2 superfamily of ubiquitin-conjugating enzymes: Constitutively active or activated through phosphorylation in the catalytic cleft. Sci. Rep..

[33] Hormaechea-Agulla D., Kim Y., Song M.S., Song S.J. (2018). New Insights into the Role of E2s in the Pathogenesis of Diseases: Lessons Learned from UBE2O. Mol. Cells.

[34] Hosseini S.M., Okoye I., Chaleshtari M.G., Hazhirkarzar B., Mohamadnejad J., Azizi G., Hojjat-Farsangi M., Mohammadi H., Shotorbani S.S., Jadidi-Niaragh F. (2019). E2 ubiquitin-conjugating enzymes in cancer: Implications for immunotherapeutic interventions. Clin. Chim. Acta..

[35] Gorlov I., Orlow I., Ringelberg C., Hernando E., Ernstoff M.S., Cheng C., Her S., Parker J.S., Thompson C.L., Gerstenblith M.R. (2018). Identification of gene expression levels in primary melanoma associated with clinically meaningful characteristics. Melanoma Res..

[36] Ueki T., Park J.-H., Nishidate T., Kijima K., Hirata K., Nakamura Y., Katagiri T. (2009). Ubiquitination and downregulation of BRCA1 by ubiquitin-conjugating enzyme E2T overexpression in human breast cancer cells. Cancer Res..

[37] Hu W., Xiao L., Cao C., Hua S., Wu D. (2016). UBE2T promotes nasopharyngeal carcinoma cell proliferation, invasion, and metastasis by activating the AKT/GSK3β/β-catenin pathway. Oncotarget.

[38] Zhang W., Zhang Y., Yang Z., Liu X., Yang P., Wang J., Hu K., He X., Zhang X., Jing H. (2019). High expression of UBE2T predicts poor prognosis and survival in multiple myeloma. Cancer Gene Ther..

[39] Dikshit A., Jin Y.J., Degan S., Hwang J., Foster M.W., Li C.-Y., Zhang J.Y. (2018). UBE2N Promotes Melanoma Growth via MEK/FRA1/SOX10 Signaling. Cancer Res..

[40] Kraft S., Moore J.B., Muzikansky A., Scott K.L., Duncan L.M. (2017). Differential UBE2C and HOXA1 expression in melanocytic nevi and melanoma. J. Cutan. Pathol..

[41] Liu G., Zhao J., Pan B., Ma G., Liu L. (2019). UBE2C overexpression in melanoma and its essential role in G2/M transition. J. Cancer.

[42] Cappadocia L., Lima C.D. (2018). Ubiquitin-like Protein Conjugation: Structures, Chemistry, and Mechanism. Chem. Rev..

[43] Mo Y.Y., Moschos S.J. (2005). Targeting Ubc9 for cancer therapy. Expert Opin. Ther. Targets.

[44] Moschos S.J., Mo Y.-Y. (2006). Role of SUMO/Ubc9 in DNA damage repair and tumorigenesis. J. Mol. Histol..

[45] Moschos S.J., Smith A.P., Mandic M., Athanassiou C., Watson-Hurst K., Jukic D.M., Edington H.D., Kirkwood J.M., Becker D. (2007). SAGE and antibody array analysis of melanoma-infiltrated lymph nodes: Identification of Ubc9 as an important molecule in advanced-stage melanomas. Oncogene.

[46] Xu W., Gong L., Haddad M.M., Bischof O., Campisi J., Yeh E.T.H., Medrano E.E. (2000). Regulation of microphthalmia-associated transcription factor MITF protein levels by association with the ubiquitin-conjugating enzyme hUBC9. Exp. Cell Res..

[47] Cheli Y., Giuliano S., Guiliano S., Botton T., Rocchi S., Hofman V., Hofman P., Bahadoran P., Bertolotto C., Ballotti R. (2011). Mitf is the key molecular switch between mouse or human melanoma initiating cells and their differentiated progeny. Oncogene.

[48] Bertolotto C., Lesueur F., Giuliano S., Strub T., De Lichy M., Bille K., Dessen P., D’Hayer B., Mohamdi H., Remenieras A. (2011). A SUMOylation-defective MITF germline mutation predisposes to melanoma and renal carcinoma. Nature.

[49] Wang P., Li Y., Ma Y., Zhang X., Li Z., Yu W., Zhu M., Wang J., Xu Y., Xu A. (2021). Comprehensive Investigation into the Role of Ubiquitin-Conjugating Enzyme E2S in Melanoma Development. J. Invest. Dermatol..

[50] Gajan A., Martin C.E., Kim S., Joshi M., Michelhaugh S.K., Sloma I., Mittal S., Firestine S., Shekhar M.P.V. (2019). Alternative Splicing of RAD6B and Not RAD6A is Selectively Increased in Melanoma: Identification and Functional Characterization. Cells.

[51] Sarma A., Gajan A., Kim S., Gurdziel K., Mao G., Nangia-Makker P., Shekhar M.P.V. (2020). RAD6B loss disrupts expression of melanoma phenotype in part by inhibiting WNT/beta-catenin signaling. Am. J. Pathol..

[52] Berndsen C.E., Wolberger C. (2014). New insights into ubiquitin E3 ligase mechanism. Nat. Struct. Mol. Biol..

[53] Lin J., Ji A., Qiu G., Feng H., Li J., Li S., Zou Y., Cui Y., Song C., He H. (2018). FBW7 is associated with prognosis, inhibits malignancies and enhances temozolomide sensitivity in glioblastoma cells. Cancer Sci..

[54] Zhang Y., Zhang X., Ye M., Jing P., Xiong J., Han Z., Kong J., Li M., Lai X., Chang N. (2018). FBW7 loss promotes epithelial-to-mesenchymal transition in non-small cell lung cancer through the stabilization of Snail protein. Cancer Lett..

[55] Shimizu K., Nihira N.T., Inuzuka H., Wei W. (2018). Physiological functions of FBW7 in cancer and metabolism. Cell. Signal..

[56] Aydin I.T., Melamed R.D., Adams S.J., Castillo-Martin M., Demir A., Bryk D., Brunner G., Cordon-Cardo C., Osman I., Rabadan R. (2014). FBXW7 mutations in melanoma and a new therapeutic paradigm. J. Natl. Cancer Inst..

[57] Saei A., Palafox M., Benoukraf T., Kumari N., Jaynes P.W., Iyengar P.V., Muñoz-Couselo E., Nuciforo P., Cortés J., Nötzel C. (2018). Loss of USP28-mediated BRAF degradation drives resistance to RAF cancer therapies. J. Exp. Med..

[58] Wan L., Chen M., Cao J., Dai X., Yin Q., Zhang J., Song S.-J., Lu Y., Liu J., Inuzuka H. (2017). The APC/C E3 Ligase Complex Activator FZR1 Restricts BRAF Oncogenic Function. Cancer Discov..

[59] Yin Q., Wyatt C.J., Han T., Smalley K.S.M., Wan L. (2020). ITCH as a potential therapeutic target in human cancers. Semin. Cancer Biol..

[60] Yin Q., Han T., Fang B., Zhang G., Zhang C., Roberts E.R., Izumi V., Zheng M., Jiang S., Yin X. (2019). K27-linked ubiquitination of BRAF by ITCH engages cytokine response to maintain MEK-ERK signaling. Nat. Commun..

[61] Montagnani V., Maresca L., Apollo A., Pepe S., Carr R.M., Fernandez-Zapico M.E., Stecca B. (2020). E3 ubiquitin ligase PARK2, an inhibitor of melanoma cell growth, is repressed by the oncogenic ERK1/2-ELK1 transcriptional axis. J. Biol. Chem..

[62] Wang S., Wu Y., Xu Y., Tang X. (2019). miR-10b promoted melanoma progression through Wnt/β-catenin pathway by repressing ITCH expression. Gene.

[63] Cheng Y., Chen G., Martinka M., Ho V., Li G. (2013). Prognostic significance of Fbw7 in human melanoma and its role in cell migration. J. Investig. Dermatol..

[64] Rambow F., Job B., Petit V., Gesbert F., Delmas V., Seberg H., Meurice G., Van Otterloo E., Dessen P., Robert C. (2015). New Functional Signatures for Understanding Melanoma Biology from Tumor Cell Lineage-Specific Analysis. Cell Rep..

[65] Nihal M., Wood G.S. (2016). c-CBL regulates melanoma proliferation, migration, invasion and the FAK-SRC-GRB2 nexus. Oncotarget.

[66] Torrino S., Visvikis O., Doye A., Boyer L., Stefani C., Munro P., Bertoglio J., Gacon G., Mettouchi A., Lemichez E. (2011). The E3 ubiquitin-ligase HACE1 catalyzes the ubiquitylation of active Rac1. Dev. Cell.

[67] Goka E.T., Lippman M.E. (2015). Loss of the E3 ubiquitin ligase HACE1 results in enhanced Rac1 signaling contributing to breast cancer progression. Oncogene.

[68] Gao Z.F., Wu Y.N., Bai Z.T., Zhang L., Zhou Q., Li X. (2016). Tumor-suppressive role of HACE1 in hepatocellular carcinoma and its clinical significance. Oncol. Rep..

[69] Zhou Z., Zhang H.-S., Zhang Z.-G., Sun H.-L., Liu H.-Y., Gou X.-M., Yu X.-Y., Huang Y.-H. (2019). Loss of HACE1 promotes colorectal cancer cell migration via upregulation of YAP1. J. Cell. Physiol..

[70] El-Hachem N., Habel N., Naiken T., Bzioueche H., Cheli Y., Beranger G.E., Jaune E., Rouaud F., Nottet N., Reinier F. (2018). Uncovering and deciphering the pro-invasive role of HACE1 in melanoma cells. Cell Death Differ..

[71] Zheng Y., Chen H., Zhao Y., Zhang X., Liu J., Pan Y., Bai J., Zhang H. (2020). Knockdown of FBXO22 inhibits melanoma cell migration, invasion and angiogenesis via the HIF-1α/VEGF pathway. Investig. New Drugs.

[72] Wu B., Liu Z.-Y., Cui J., Yang X.-M., Jing L., Zhou Y., Chen Z.-N., Jiang J.-L. (2017). F-Box Protein FBXO22 Mediates Polyubiquitination and Degradation of CD147 to Reverse Cisplatin Resistance of Tumor Cells. Int. J. Mol. Sci..

[73] Habel N., El-Hachem N., Soysouvanh F., Hadhiri-Bzioueche H., Giuliano S., Nguyen S., Horák P., Gay A.-S., Debayle D., Nottet N. (2021). FBXO32 links ubiquitination to epigenetic reprograming of melanoma cells. Cell Death Differ..

[74] Li F.Z., Dhillon A.S., Anderson R.L., McArthur G., Ferrao P.T. (2015). Phenotype Switching in Melanoma: Implications for Progression and Therapy. Front. Oncol..

[75] Song Y., Lin M., Liu Y., Wang Z.W., Zhu X. (2019). Emerging role of F-box proteins in the regulation of epithelial-mesenchymal transition and stem cells in human cancers. Stem Cell Res. Ther..

[76] He H., Dai J., Xu Z., He W., Wang X., Zhu Y., Wang H. (2018). Fbxw7 regulates renal cell carcinoma migration and invasion via suppression of the epithelial-mesenchymal transition. Oncol. Lett..

[77] Li Q., Murphy M., Ross J., Sheehan C., Carlson J.A. (2004). Skp2 and p27kip1 expression in melanocytic nevi and melanoma: An inverse relationship. J. Cutan. Pathol..

[78] Liu S., Yamauchi H. (2009). p27-Associated G1 arrest induced by hinokitiol in human malignant melanoma cells is mediated via down-regulation of pRb, Skp2 ubiquitin ligase, and impairment of Cdk2 function. Cancer Lett..

[79] Katagiri Y., Hozumi Y., Kondo S. (2006). Knockdown of Skp2 by siRNA inhibits melanoma cell growth in vitro and in vivo. J. Dermatol. Sci..

[80] Zhao H., Pan H., Wang H., Chai P., Ge S., Jia R., Fan X. (2019). SKP2 targeted inhibition suppresses human uveal melanoma progression by blocking ubiquitylation of p27. Oncol. Targets. Ther..

[81] Qu X., Shen L., Zheng Y., Cui Y., Feng Z., Liu F., Liu J. (2014). A signal transduction pathway from TGF-β1 to SKP2 via Akt1 and c-Myc and its correlation with progression in human melanoma. J. Investig. Dermatol..

[82] Larue L., Bellacosa A. (2005). Epithelial-mesenchymal transition in development and cancer: Role of phosphatidylinositol 3’ kinase/AKT pathways. Oncogene.

[83] Wei C.-Y., Zhu M.-X., Yang Y.-W., Zhang P.-F., Yang X., Peng R., Gao C., Lu J.-C., Wang L., Deng X.-Y. (2019). Downregulation of RNF128 activates Wnt/β-catenin signaling to induce cellular EMT and stemness via CD44 and CTTN ubiquitination in melanoma. J. Hematol. Oncol..

[84] Dai Z., Zhang J., Wu Q., Fang H., Shi C., Li Z., Lin C., Tang D., Wang D. (2020). Intestinal microbiota: A new force in cancer immunotherapy. Cell Commun. Signal..

[85] Li Y., Tinoco R., Elmén L., Segota I., Xian Y., Fujita Y., Sahu A., Zarecki R., Marie K., Feng Y. (2019). Gut microbiota dependent anti-tumor immunity restricts melanoma growth in Rnf5 −/− mice. Nat. Commun..

[86] Nicholson B., Leach C.A., Goldenberg S.J., Francis D.M., Kodrasov M.P., Tian X., Shanks J., Sterner D.E., Bernal A., Mattern M.R. (2008). Characterization of ubiquitin and ubiquitin-like-protein isopeptidase activities. Protein Sci..

[87] McClurg U.L., Robson C.N. (2015). Deubiquitinating enzymes as oncotargets. Oncotarget.

[88] Ventii K.H., Devi N.S., Friedrich K.L., Chernova T.A., Tighiouart M., Van Meir E.G., Wilkinson K.D. (2008). BRCA1-associated protein-1 is a tumor suppressor that requires deubiquitinating activity and nuclear localization. Cancer Res..

[89] Wiesner T., Obenauf A.C., Murali R., Fried I., Griewank K.G., Ulz P., Windpassinger C., Wackernagel W., Loy S., Wolf I. (2011). Germline mutations in BAP1 predispose to melanocytic tumors. Nat. Genet..

[90] Scheuermann J.C., de Ayala Alonso A.G., Oktaba K., Ly-Hartig N., McGinty R.K., Fraterman S., Wilm M., Muir T.W., Müller J. (2010). Histone H2A deubiquitinase activity of the Polycomb repressive complex PR-DUB. Nature.

[91] Yu H., Pak H., Hammond-Martel I., Ghram M., Rodrigue A., Daou S., Barbour H., Corbeil L., Hébert J., Drobetsky E. (2014). Tumor suppressor and deubiquitinase BAP1 promotes DNA double-strand break repair. Proc. Natl. Acad. Sci. USA.

[92] Webster J.D., Pham T.H., Wu X., Hughes N.W., Li Z., Totpal K., Lee H.-J., Calses P.C., Chaurushiya M.S., Stawiski E.W. (2019). The tumor suppressor BAP1 cooperates with BRAFV600E to promote tumor formation in cutaneous melanoma. Pigment Cell Melanoma Res..

[93] He M., Chaurushiya M.S., Webster J.D., Kummerfeld S., Reja R., Chaudhuri S., Chen Y.-J., Modrusan Z., Haley B., Dugger D.L. (2019). Intrinsic apoptosis shapes the tumor spectrum linked to inactivation of the deubiquitinase BAP1. Science.

[94] Lee H.-J., Pham T., Chang M.T., Barnes D., Cai A.G., Noubade R., Totpal K., Chen X., Tran C., Hagenbeek T. (2020). The tumor suppressor BAP1 regulates the Hippo pathway in pancreatic ductal adenocarcinoma. Cancer Res..

[95] Liu-Smith F., Lu Y. (2020). Opposite Roles of BAP1 in Overall Survival of Uveal Melanoma and Cutaneous Melanoma. J. Clin. Med..

[96] Kumar R., Taylor M., Miao B., Ji Z., Njauw J.C.-N., Jönsson G., Frederick D.T., Tsao H. (2015). BAP1 has a survival role in cutaneous melanoma. J. Invest. Dermatol..

[97] Sato Y., Goto E., Shibata Y., Kubota Y., Yamagata A., Goto-Ito S., Kubota K., Inoue J., Takekawa M., Tokunaga F. (2015). Structures of CYLD USP with Met1- or Lys63-linked diubiquitin reveal mechanisms for dual specificity. Nat. Struct. Mol. Biol..

[98] Massoumi R., Podda M., Fässler R., Paus R. (2006). Cylindroma as tumor of hair follicle origin. J. Investig. Dermatol..

[99] Massoumi R., Kuphal S., Hellerbrand C., Haas B., Wild P., Spruss T., Pfeifer A., Fässler R., Bosserhoff A.K. (2009). Down-regulation of CYLD expression by Snail promotes tumor progression in malignant melanoma. J. Exp. Med..

[100] De Jel M.M., Schott M., Lamm S., Neuhuber W., Kuphal S., Bosserhoff A.-K. (2019). Loss of CYLD accelerates melanoma development and progression in the Tg(Grm1) melanoma mouse model. Oncogenesis.

[101] Young M.-J., Hsu K.-C., Lin T.E., Chang W.-C., Hung J.-J. (2019). The role of ubiquitin-specific peptidases in cancer progression. J. Biomed. Sci..

[102] Guo W., Ma J., Pei T., Zhao T., Guo S., Yi X., Liu Y., Wang S., Zhu G., Jian Z. (2018). Up-regulated deubiquitinase USP4 plays an oncogenic role in melanoma. J. Cell. Mol. Med..

[103] Zou X., Levy-Cohen G., Blank M. (2015). Molecular functions of NEDD4 E3 ubiquitin ligases in cancer. Biochim. Biophys. Acta—Rev. Cancer.

[104] Aronchik I., Kundu A., Quirit J.G., Firestone G.L. (2014). The antiproliferative response of indole-3-carbinol in human melanoma cells is triggered by an interaction with NEDD4-1 and disruption of wild-type PTEN degradation. Mol. Cancer Res..

[105] Yang Y., Luo M., Zhang K., Zhang J., Gao T., Connell D.O., Yao F., Mu C., Cai B., Shang Y. (2020). Nedd4 ubiquitylates VDAC2/3 to suppress erastin-induced ferroptosis in melanoma. Nat. Commun..

[106] Kim H., Frederick D.T., Levesque M.P., Cooper Z.A., Feng Y., Krepler C., Brill L., Samuels Y., Hayward N.K., Perlina A. (2015). Downregulation of the Ubiquitin Ligase RNF125 Underlies Resistance of Melanoma Cells to BRAF Inhibitors via JAK1 Deregulation. Cell Rep..

[107] Zhao H., Li C.C., Pardo J., Chu P.C., Liao C.X., Huang J., Dong J.G., Zhou X., Huang Q., Huang B. (2005). A novel E3 ubiquitin ligase TRAC-1 positively regulates T cell activation. J. Immunol..

[108] Arimoto K., Takahashi H., Hishiki T., Konishi H., Fujita T., Shimotohno K. (2007). Negative regulation of the RIG-I signaling by the ubiquitin ligase RNF125. Proc. Natl. Acad. Sci. USA.

[109] Avitan-Hersh E., Feng Y., Oknin Vaisman A., Abu Ahmad Y., Zohar Y., Zhang T., Lee J.S., Lazar I., Sheikh Khalil S., Feiler Y. (2020). Regulation of eIF2α by RNF4 Promotes Melanoma Tumorigenesis and Therapy Resistance. J. Invest. Dermatol..

[110] Nagler A., Vredevoogd D.W., Alon M., Cheng P.F., Trabish S., Kalaora S., Arafeh R., Goldin V., Levesque M.P., Peeper D.S. (2020). A genome-wide CRISPR screen identifies FBXO42 involvement in resistance toward MEK inhibition in NRAS-mutant melanoma. Pigment Cell Melanoma Res..

[111] Schülein-Völk C., Wolf E., Zhu J., Xu W., Taranets L., Hellmann A., Jänicke L.A., Diefenbacher M.E., Behrens A., Eilers M. (2014). Dual regulation of Fbw7 function and oncogenic transformation by Usp28. Cell Rep..

[112] Didier R., Mallavialle A., Ben Jouira R., Domdom M.A., Tichet M., Auberger P., Luciano F., Ohanna M., Tartare-Deckert S., Deckert M. (2018). Targeting the Proteasome-Associated Deubiquitinating Enzyme USP14 Impairs Melanoma Cell Survival and Overcomes Resistance to MAPK-Targeting Therapies. Mol. Cancer Ther..

[113] Vanneste M., Feddersen C.R., Varzavand A., Zhu E.Y., Foley T., Zhao L., Holt K.H., Milhem M., Piper R., Stipp C.S. (2020). Functional Genomic Screening Independently Identifies CUL3 as a Mediator of Vemurafenib Resistance via Src-Rac1 Signaling Axis. Front. Oncol..

[114] Han Y., Liu D., Li L. (2020). PD-1/PD-L1 pathway: Current researches in cancer. Am. J. Cancer Res..

[115] Meng X., Liu X., Guo X., Jiang S., Chen T., Hu Z., Liu H., Bai Y., Xue M., Hu R. (2018). FBXO38 mediates PD-1 ubiquitination and regulates anti-tumour immunity of T cells. Nature.

[116] Saldana M., VanderVorst K., Berg A.L., Lee H., Carraway K.L. (2019). Otubain 1: A non-canonical deubiquitinase with an emerging role in cancer. Endocr. Relat. Cancer.

[117] Zhou X., Yu J., Cheng X., Zhao B., Manyam G.C., Zhang L., Schluns K., Li P., Wang J., Sun S.-C. (2019). The deubiquitinase Otub1 controls the activation of CD8+ T cells and NK cells by regulating IL-15-mediated priming. Nat. Immunol..

[118] Scortegagna M., Hockemeyer K., Dolgalev I., Poźniak J., Rambow F., Li Y., Feng Y., Tinoco R., Otero D.C., Zhang T. (2020). Siah2 control of T-regulatory cells limits anti-tumor immunity. Nat. Commun..

[119] Feng Y., Sessions E.H., Zhang F., Ban F., Placencio-Hickok V., Ma C.T., Zeng F.Y., Pass I., Terry D.B., Cadwell G. (2019). Identification and characterization of small molecule inhibitors of the ubiquitin ligases Siah1/2 in melanoma and prostate cancer cells. Cancer Lett..

[120] Mezzadra R., Sun C., Jae L.T., Gomez-Eerland R., De Vries E., Wu W., Logtenberg M.E.W., Slagter M., Rozeman E.A., Hofland I. (2017). Identification of CMTM6 and CMTM4 as PD-L1 protein regulators. Nature.

[121] Merin N., Kelly K. (2014). Clinical Use of Proteasome Inhibitors in the Treatment of Multiple Myeloma. Pharmaceuticals.

[122] Thompson J.L. (2013). Carfilzomib: A second-generation proteasome inhibitor for the treatment of relapsed and refractory multiple myeloma. Ann. Pharmacother..

[123] Richardson P.G., Zweegman S., O’Donnell E.K., Laubach J.P., Raje N., Voorhees P., Ferrari R.H., Skacel T., Kumar S.K., Lonial S. (2018). Ixazomib for the treatment of multiple myeloma. Expert Opin. Pharmacother..

[124] Markovic S.N., Geyer S.M., Dawkins F., Sharfman W., Albertini M., Maples W., Fracasso P.M., Fitch T., Lorusso P., Adjei A.A. (2005). A phase II study of bortezomib in the treatment of metastatic malignant melanoma. Cancer.

[125] Bhatia S., Pavlick A.C., Boasberg P., Thompson J.A., Mulligan G., Pickard M.D., Faessel H., Dezube B.J., Hamid O. (2016). A phase I study of the investigational NEDD8-activating enzyme inhibitor pevonedistat (TAK-924/MLN4924) in patients with metastatic melanoma. Investig. New Drugs.

[126] Wertz I.E., Wang X. (2019). From Discovery to Bedside: Targeting the Ubiquitin System. Cell Chem. Biol..

[127] Pawlak W.Z., Legha S.S. (2004). Phase II study of thalidomide in patients with metastatic melanoma. Melanoma Res..

[128] Glaspy J., Atkins M.B., Richards J.M., Agarwala S.S., O’Day S., Knight R.D., Jungnelius J.U., Bedikian A.Y. (2009). Results of a multicenter, randomized, double-blind, dose-evaluating phase 2/3 study of lenalidomide in the treatment of metastatic malignant melanoma. Cancer.

[129] Altun M., Kramer H.B., Willems L.I., McDermott J.L., Leach C.A., Goldenberg S.J., Kumar K.G.S., Konietzny R., Fischer R., Kogan E. (2011). Activity-Based Chemical Proteomics Accelerates Inhibitor Development for Deubiquitylating Enzymes. Chem. Biol..

[130] Sun X., Gao H., Yang Y., He M., Wu Y., Song Y., Tong Y., Rao Y. (2019). PROTACs: Great opportunities for academia and industry. Signal Transduct. Target. Ther..

[131] Vogelmann A., Robaa D., Sippl W., Jung M. (2020). Proteolysis targeting chimeras (PROTACs) for epigenetics research. Curr. Opin. Chem. Biol..

